# Recent Advancements in 3D Printing of Polysaccharide Hydrogels in Cartilage Tissue Engineering

**DOI:** 10.3390/ma14143977

**Published:** 2021-07-16

**Authors:** Jakob Naranda, Matej Bračič, Matjaž Vogrin, Uroš Maver

**Affiliations:** 1 Department of Orthopaedics, University Medical Centre Maribor, SI-2000 Maribor, Slovenia; Matjaz.VOGRIN@ukc-mb.si; 2 Faculty of Mechanical Engineering, University of Maribor, SI-2000 Maribor, Slovenia; matej.bracic@um.si; 3 Department of Orthopaedics, Faculty of Medicine, University of Maribor, SI-2000 Maribor, Slovenia; 4 Institute of Biomedical Sciences, Faculty of Medicine, University of Maribor, SI-2000 Maribor, Slovenia; 5 Department of Pharmacology, Faculty of Medicine, University of Maribor, SI-2000 Maribor, Slovenia

**Keywords:** cartilage tissue engineering, hydrogels, polysaccharides, proteins, proteo-saccharides, 3D (bio)printing

## Abstract

The application of hydrogels coupled with 3-dimensional (3D) printing technologies represents a modern concept in scaffold development in cartilage tissue engineering (CTE). Hydrogels based on natural biomaterials are extensively used for this purpose. This is mainly due to their excellent biocompatibility, inherent bioactivity, and special microstructure that supports tissue regeneration. The use of natural biomaterials, especially polysaccharides and proteins, represents an attractive strategy towards scaffold formation as they mimic the structure of extracellular matrix (ECM) and guide cell growth, proliferation, and phenotype preservation. Polysaccharide-based hydrogels, such as alginate, agarose, chitosan, cellulose, hyaluronan, and dextran, are distinctive scaffold materials with advantageous properties, low cytotoxicity, and tunable functionality. These superior properties can be further complemented with various proteins (e.g., collagen, gelatin, fibroin), forming novel base formulations termed “proteo-saccharides” to improve the scaffold’s physiological signaling and mechanical strength. This review highlights the significance of 3D bioprinted scaffolds of natural-based hydrogels used in CTE. Further, the printability and bioink formation of the proteo-saccharides-based hydrogels have also been discussed, including the possible clinical translation of such materials.

## 1. Introduction

Articular cartilage is characterized by a limited intrinsic regenerative capacity after injury. Recent research efforts showed that cartilage tissue engineering (CTE) is still one of the most promising fields of research, but is also plagued by considerable unsolved challenges that hinder its implementation in clinical practice [1]. The state-of-the-art concept of cartilage tissue development combines the use of biocompatible and biodegradable three-dimensional (3D) bio-materials (scaffolds), providing initial support for the desired cells to attach, proliferate, and form their native extracellular matrix (ECM). Scaffolds used for this purpose must mimic the native tissue at the bio-chemo-mechanical level and, together with the application of growth factors (GF) and mechanical stimulation, promote growth and direct differentiation towards the desired chondrocyte phenotype [2]. The ultimate goal of cartilage tissue engineering is to create artificial matrices that can be used in in vivo applications (e.g., in cartilage joint repair) [3].

One of the major challenges in CTE is selecting appropriate materials and processing strategies for scaffold preparation that preserve the chondrocyte phenotype and allow functional cartilage tissue maturation [4]. Scaffold development has to be based on biocompatible and biodegradable materials. Simultaneously, the manufacturing technique must allow the design of supports of different shapes and sizes with a controlled microstructure [5]. Currently, polymeric scaffolds, especially hydrogels, have attracted particular interest because they allow for a versatile range of CTE variations [6]. An interesting approach to scaffold formation is using hydrogels as biomaterials and 3D printing or rapid prototyping (RP) technologies for fabrication [7]. The combined potential of hydrogels and 3D printing technologies permits automated and reproducible formation of fully interconnected 3D structures with defined dimensions and architectural characteristics [8]. This allows for the fabrication of 3D structures by depositing (biocompatible) materials in a layer-by-layer approach with precise control over the internal architecture and, through proper material choice, for control over the degradation rate and mechanical strength [9,10]. Recently, biofabrication (bioprinting), involving the processing of functional biological constructs combined with living cells (bioink), is being increasingly applied in CTE [11,12].

Various materials, either natural or synthetic, or both, have been used to make hydrogel-based scaffolds. Synthetic polymers are an attractive choice for scaffold formation because of their reproducible properties, controllable degradation, and mechanical properties. However, due to their low bioactivity and biocompatibility, their translation into clinical practice is limited. Therefore, degradable biopolymers of natural origin, similar to the components present in cartilage ECM, have been widely used as CTE biomaterials [13]. They are readily accepted by the body and possess outstanding biocompatibility, low immunological response, low cytotoxicity, and excellent capability to promote cell adhesion, proliferation, and regeneration of new tissues [14]. Naturally derived hydrogels used in CTE may be protein-based (gelatin, collagen, fibroin, etc.), polysaccharide-based (alginate, agarose, chitosan, cellulose, hyaluronic acid (HA), dextran) or are made from decellularized tissue [15]. Polysaccharides are distinctive scaffold materials, and their advantageous properties, such as biocompatibility, biodegradability, and tunable functionality, raise them among the most favorable scaffold materials. Further, polysaccharides-based hydrogels may represent distinct similarities to the cartilage ECM, making them excellent scaffold material for CTE use [16].

An important concern with using allogeneic and xenogeneic materials is the risk of zoonoses due to the transmission of bacteria, viruses, and prions, as well as potential immunological reactions related to their foreign origin [17]. Traditionally, commercialized collagens have been extracted mainly from terrestrial mammals, such as cattle and pigs, and are widely used in the food, cosmetic, pharmaceutical, and biomedical industries [18]. The high risk of prion transmission associated with mammal-derived collagen has prompted research into its alternative sources, such as marine-origin collagen [19]. However, new applications often focus on other source materials, such as natural biomacromolecules, decellularized extracellular matrix (ECM) scaffolds, and autologous preparations rich in growth factors [17].

**Figure 1:**
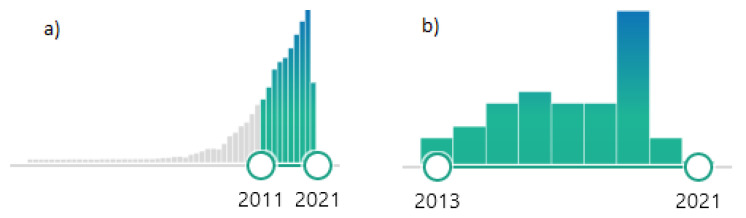
Number of publications in Pubmed (search performed in April 2021): (**a**) keywords: “polysaccharides hydrogels” (**b**) keywords: “cartilage tissue engineering, hydrogel, polysaccharide, 3D printing”.

This review discusses the developmental and biological aspects of 3D bioprinted scaffolds of various important natural polysaccharide hydrogels widely used in CTE (alginate, agarose, chitosan, cellulose, HA, dextran). The printability and bioink formation of the polysaccharides-based hydrogel scaffolds and their combination with proteins (gelatin, collagen, fibroin, etc.) are discussed. Future perspectives of 3D printing of polysaccharide–protein-based hydrogels are outlined, and their possible clinical translation is evaluated. Although there are already many studies dedicated to the investigation of hydrogel-based 3D printing and 3D bioprinting, a comprehensive review in the field of 3D printing of polysaccharide protein-based hydrogels in CTE has not yet been published. As seen from the number of publications on polysaccharide hydrogels, the interest in such materials and their application increased tremendously in recent times. The publishing of more than 7000 publications in Pubmed on this topic in the last ten years indicate that this is an extremely attractive research field (Figure 1a). The number of publications using the keywords of this review article (cartilage tissue engineering, hydrogel, polysaccharide, 3D printing) generated only 36 items in Pubmed (Figure 1b). Among these, only three review articles were identified, which, however, do not address the topic of this review article.

## 2. Materials and Methods

A literature review was conducted via the biggest medical literature databases (Medline, PubMed, ScienceDirect) to obtain studies related to articular cartilage regeneration. The employed search terms in the form of keywords were “cartilage tissue engineering”, “3D-(bio)printing”, “polysaccharides hydrogel”, “bioinks”. The used Medical Subject Headings (MeSH) identifiers were “articular cartilage”, “tissue engineering”, “bioprinting”, “polysaccharides”, “hydrogels”, “tissue scaffolds”. With the help of this search algorithm and specific filters (5-year, review), we were able to find relevant new impactful studies on articular cartilage regeneration, which were included in this review.

## 3. 3D Printing of Hydrogels in CTE

Biomaterial-based hydrogels are among the most promising materials for various tissue engineering applications since they can be engineered into almost any shape and size. They can also be functionalized into hydrogel composites, giving them improved properties tailored to specific applications, for example, CTE [20,21]. In general, they represent hydrophilic 3D networks composed of water-soluble natural (polysaccharides and proteins) and/or synthetic polymers crosslinked by chemical or physical methods to form a water-insoluble hydrogel [22]. In recent years, polymeric hydrogels and hydrogel composites have been intensively studied due to their ability to facilitate retention, adhesion, migration, proliferation, and cellular differentiation. Hydrogels have been proven to provide highly desirable 3D environments for the regeneration of cartilaginous tissue, as shown in in vitro and in vivo studies [23,24].

Hydrogels are considered unique viscoelastic materials, whereas their elastic modulus and viscosity are often determined to characterize their mechanical strength. The mechanical properties of the scaffolds used in cartilage tissue engineering (CTE) are of utmost importance since they should resemble the properties of the articular cartilage and simulate the conditions in the native environment (e.g., provided through movement) [25]. In general, it is essential to adjust the mechanical properties of an artificial construct to the desired tissue: for soft tissue, a strength between 0.4 and 350 MPa is desired, while for hard tissue regeneration, an intensity of 10–1500 Mpa should be achieved [26]. Cartilage tissue is somewhat extra demanding in this sense. Namely, it exhibits a dynamic modulus from 0.1 MPa to 3 MPa over the frequency range of 1–1000 Hz, encompassing most daily life activities [27].

Natural polysaccharide-based scaffolds used in CTE reach compressive Young’s moduli values between 10 and 250 kPa [28,29,30], corresponding to the low end of ranges reported for native knee cartilage. On the other side of mechanical demands, some polysaccharide-based materials also reach strengths up to 30 Mpa [31,32]. Such values are very well matching synthetic materials, such as polylactic acid (PLA), poly(D,L-lactide-co-glycolide (PLGA), poly-L-lactide (PLLA), and similar [33,34].

The mechanical strength of the scaffold is inversely related to the rate of degradation. Therefore, these two opposite properties should be balanced through optimal design. More information related to this part is added in the following answer.

The 3D bioprinting of hydrogels has attracted much attention in the field of tissue engineering. Although 3D bioprinting of polymeric hydrogels in CTE is proving to be extremely promising, there are additional challenges to be overcome. These include selecting appropriate biomaterials for bioink fabrication, the cell types to be incorporated, the inclusion of bioactive molecules to promote maintenance of the desired phenotype, mechanical properties that promote cell growth, and the technical challenges of fabricating complex scaffold geometries with living cells [35,36]. A chief concern with 3D printing of cell-laden hydrogels is optimizing the printing parameters to enable/preserve control over material properties and promote efficient printing while maintaining cell viability and phenotype [37]. Nowadays, hydrogel systems and cell encapsulation strategies are commonly used to fabricate 3D tissue constructs [38,39]. Simultaneous and sustained presentation of regenerative molecules in the hydrogels offer more benefits since these could initiate and enhance the survival rate, proliferation, differentiation, and ECM production of co-encapsulated cells [37]. The most recent advancement represents the use of intelligent materials that may change their shape, produce an electrical current, become bioactive, or perform an intended function in response to an external stimulus, termed dynamic 3D structures or 4D printing [40].

In general, hydrogel 3D printing can be divided into three main methods: laser-based, nozzle-based, and inkjet-based systems. During processing, the hydrogel is transferred from the reservoir to an ejection system, followed by extrusion onto a collection platform in a controlled manner. Nozzle and inkjet printers deposit material sequentially, whereas laser-assisted systems are based on the pre-deposited material’s photopolymerization in a specific predefined pattern [7]. The scaffold’s fabrication using the 3D bioprinting technique requires a specific set of properties of the bioink to create an appropriate 3D bioprinted structure [41]. An ideal bioink possesses proper mechanical, rheological, and biological properties of the target tissue. It is essential to ensure correct functionality, cell function, and regeneration of the bioprinted tissue [35,36]. The lack of suitable bioinks restricts the progress of tissue regeneration and its translation into clinical practice [42]. Two major types of bioink materials have been used in the bioprinting of 3D tissue: the most common scaffold-based bioink, where cells are loaded in the hydrogel or other biomaterials, and scaffold-free bioink, where cells are bioprinted without the use of an exogenous biomaterial [9]. The current approach of 3D printing of hydrogel scaffold in CTE can be performed directly from hydrogels or composite hydrogels. The advantage of using unique hydrogels is based on a simpler printability process compared to hybrid bioprinting. On the other hand, composite hydrogels can offer superior mechanical performance to support the 3D structure, although it may reduce the bioactivity [32].

### 3.1. Polysaccharides-Based Hydrogels for 3D Printing in CTE

**Figure 2:**
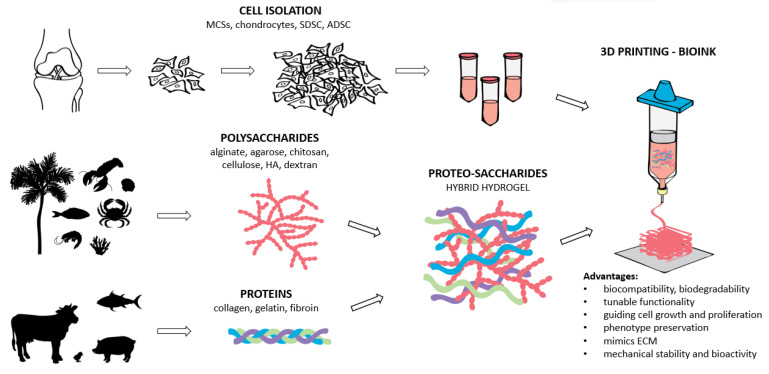
The schematic overview of the proteo-saccharides combination, their origin, and bioink preparation for hydrogel formation in CTE.

Biomacromolecules (polysaccharides and proteins), derived from both animals and plants, are the top base material choice in biomaterial science and scaffold development. Scaffold research has progressed due to polysaccharides (e.g., alginate, agarose, chitosan, cellulose, dextran, and hyaluronic acid—HA) with excellent properties concerning various tissue engineering applications [32]. These include their abundance in nature, dynamic bonds, hydrophilicity, biocompatibility, biodegradability, and the presence of functional groups, allowing for further functionalization [43]. Polysaccharide-based hydrogels were shown to be promising materials for drug delivery systems [44] and tissue engineering of soft tissues and cartilage [16,43]. They are constructed via crosslinking of polysaccharide derivatives with various functional molecules. These hydrogels often exhibit physical–chemical properties that are very different from macromolecular constituents’ properties in terms of mechanical strength, limited elongation or reproducibility, and poor toughness [45]. Superior mechanical properties, physiological signaling, and favorable tissue response can be achieved through chemical modification of polysaccharides. However, the modification of polysaccharides is often time-consuming and requires extensive chemical reactions. To improve the scaffold’s physiological signaling and mechanical strength, polysaccharides can be combined with proteins [46]. This combination, termed proteo-saccharides, represents an attractive strategy for scaffold formation in CTE as it mimics the structure of ECM and guides cell growth, proliferation, and phenotype preservation [47]. In addition, proteo-saccharides are more stable and bioactive than their parent polymers, and natural proteo-saccharides are preferred due to their reduced toxicity. A schematic overview of proteo-saccharides preparation and use, including their origin and hydrogel preparation for 3D printing in CTE is shown in Figure 2. Among natural proteo-saccharides, the following were employed as important bioink materials in CTE: gelatin–alginate, collagen–alginate, fibrin–alginate, fibrin–agarose, and others (Table 1). Furthermore, polysaccharides are interesting biomaterials since they can be combined to form composite polysaccharides’ hydrogels termed polysaccharide hybrids, for example, alginate–nanocellulose, alginate–methylcellulose (MC), HA–agarose, etc. [46,47,48]. Hence, hybrid polysaccharide hydrogels in combination with proteins give a whole new perspective in the development of a suitable scaffold and its applications in bioprinting as bioinks for CTE. Polysaccharide-based hydrogels in combination with proteins (proteosaccharides) for CTE were mainly investigated in vitro with an average culturing period between 3–4 weeks [49,50], 5 weeks [51], or 8 weeks [52]. The in vivo study using gelatin/hyaluronic acid hybrid scaffold for cartilage and subchondral bone regeneration of a rabbit femoral condyle lasted 12 weeks. Hybrid scaffolds with different collagen concentrations were implanted into cartilage defects of rabbit ears for 6 months [53].

In the following sections, we focus on 3D printing of polysaccharide hydrogels for CTE, which have shown the most promise for such an application. In addition, we highlight possible combinations with proteins for better scaffold functionality and properties by exploiting the properties of both types of macromolecules.

#### 3.1.1. Alginate Hydrogels for CTE

Alginate, a natural polysaccharide, is one of the most commonly employed materials in 3D bioprinting [54]. It is a water-soluble polysaccharide derived from brown algae or bacteria and is composed of α-L-guluronate (G blocks) and (1,4)-linked β-D-mannuronate (M blocks). The ratio of G/M blocks defines the viscosity and mechanical strength of the formed hydrogel [55]. Alginate as a biomaterial has found numerous biomedical applications due to its advantageous properties such as biocompatibility, low immunological response, degradability, process flexibility, and excellent printability. Since it can form gels under benign conditions, it is also attractive for cell encapsulation [32]. Current 3D printing techniques for the alginate-based bioinks are extrusion-assisted bioprinting and inkjet-assisted bioprinting [56]. During the process of alginate preparation, the structure and shape fidelity is difficult to guarantee. Usually, alginate-based hydrogels possess inferior mechanical properties that may affect their ability to maintain the scaffold’s architecture and the regenerated tissue structure [57]. Besides, alginate sometimes demonstrates minimal cellular adhesion and a variable degradation rate. The latter results from its often-used ionic crosslinking approach (mostly through cations like Ca^2+^), which can be exchanged during exposure to Na^+^-rich media (e.g., PBS). Despite the general good biocompatibility, this instability can lead to poor cell differentiation and cell proliferation on alginate. Hence, growth factors (e.g., transforming growth factor beta (TGF beta)) [58,59] and adhesion peptides attached to alginate bioinks have been applied to enhance cellular adhesion and cellular growth [60,61].

**Figure 3:**
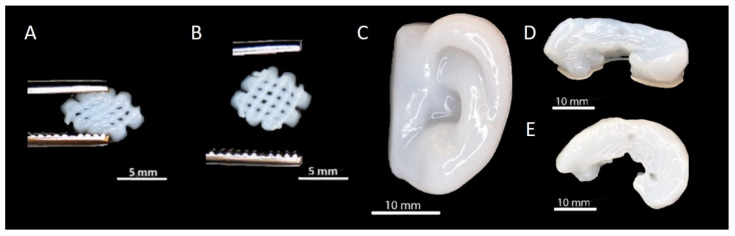
The 3D printed structures from nanocellulose-reinforced alginate hydrogels: (**A**) Small grid squeezed with tweezers, (**B**) grid recovery after squeezing, (**C**) human ear, (**D**) side view of a sheep meniscus and (**E**) top view of a sheep meniscus [66].

Remarkable improvements have been made regarding the alginate-based hydrogels’ mechanical requirements for CTE by combining alginate with inorganic fillers or other natural and/or synthetic biopolymers [54]. A variety of combinations with alginate were introduced in regenerative medicine and CTE. For example, fibrin and alginate hydrogels have been widely used to support chondrogenesis of bone marrow-derived mesenchymal stem cells (BM-MSCs) to form articular cartilage [62]. The combination of gelatin and alginate bioinks was recently introduced for cell-friendly and facile fabrication of cell-laden hydrogel constructs [46,63]. The 3D printed composite gelatin/alginate hydrogel scaffold reinforced with nano-hydroxyapatite (n-HAP) showed no cytotoxicity and supported the adhesion and growth of mouse chondrocytes [31]. An innovative bioink combining alginate, gelatin, and fibrinogen was shown promising for 3D bioprinting by the extrusion of an original bioink containing a low concentration of MSCs [49]. The alginate was also used as a coating of fibrin and HA composite gels for rabbit chondrocytes’ cartilage formation [50]. Furthermore, collagen–alginate [64] and alginate–nanocellulose [65,66] bioinks were supported for 3D cell printing in CTE (Figure 3).

#### 3.1.2. Agarose Hydrogels for CTE

**Figure 4:**
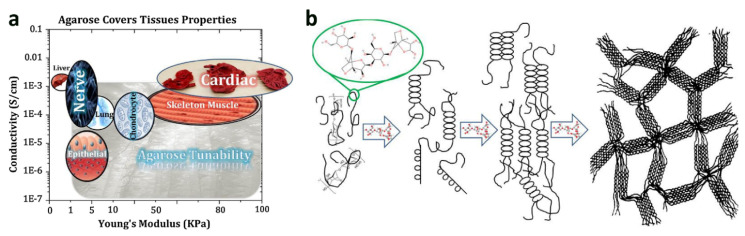
Agarose-based scaffold: (**a**) Adjustable features of agarose can result in flexible characteristics, (**b**) the molecular structure of agarose and schematic of its gelling process [67].

Agarose, found in red algae, is a water-soluble linear polysaccharide and one of agar’s main constituents. Due to its biocompatibility, controlled self-gelling properties, water solubility, and adaptable mechanical properties, it has been widely used in biomedical applications [67] (Figure 4). Agarose-based hydrogels can support cellular adhesion, proliferation, and activity owing to their stiffness and functional groups [68]. Besides this, agarose-based scaffolds were shown suitable for the bioprinting material in tissue engineering [29] and have considerably been applied in CTE to preserve the chondrocyte phenotype and enhance cartilage ECM deposition [28,69]. To enhance the cellular activity, fibroin/agarose blends were shown as an alternative biomaterial for CTE [70]. Similarly, encapsulating human elastic cartilage-derived chondrocytes (HECDC) in biodegradable nanostructured fibrin–agarose hydrogels were used to generate biodegradable and biologically active constructs for CTE applications [51].

#### 3.1.3. Chitosan Hydrogels for CTE

**Figure 5:**
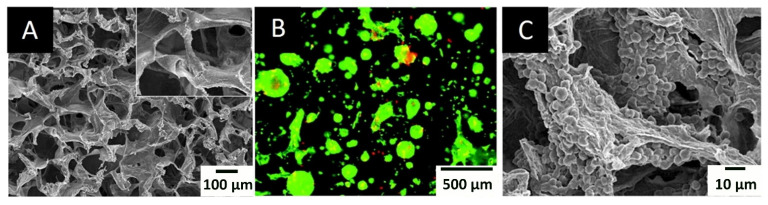
SEM (**A**,**B**) and fluorescence microscopy image (**B**) of a chitosan–alginate scaffold (**A**) and chondrocyte cells grown on it (**B**,**C**) [84].

Chitosan, obtained by deacetylation of chitin found in shrimp, crab, and coral, is a polymer composed of D-glucosamine and N-acetylglucosamine. Chitosan has been used for cartilage repair due to its intrinsic properties such as biocompatibility, biodegradability, low immunological response, and similar properties. It is often used together with glycosaminoglycans (GAGs), which are among the main constituents of ECM in cartilage tissue [81]. Furthermore, chitosan can regulate cellular fate processes by interacting with growth factors and cytokines [82]. A wide range of possibilities for combining chitosan with other materials in chitosan-based hydrogels has already been used in CTE. These combinations include natural (e.g., collagen, gelatin, alginate, hyaluronic acid, silk fibroin), chondroitin sulfate, and synthetic (e.g., polycaprolactone and polylactic acid, polyvinyl alcohol) polymers, as well as bioceramics (e.g., calcium phosphate, calcium polyphosphate, and hydroxyapatite) [30,81,82,83,84,85,86,87]. Chitosan is also used frequently in combination with anionic polysaccharides to form ion polysaccharide complexes. Such hydrogels promise easy preparation without the need to use an additional crosslinker and multifunctionality, combining properties of both types of polysaccharides in the complex [87], but mostly the cell compatibility of chitosan, as one of its major drawbacks can be improved. Chitosan ion complexes with chondroitin sulfates [88] and hyaluronic acid [89] demonstrated to support cell survival and chondrogenesis. The anionic polysaccharide alginate is frequently studied in ion complexes with chitosan in CTE [74,84,90]. For example, a chitosan–alginate ion complex was used in hydrogel preparation for its ability to be formed at neutral pH, allowing proteins or drugs to be incorporated uniformly with minimal denaturation and its ability to promote cell proliferation, i.e., enhance phenotype expression of HTB-94 chondrocytes, providing an upgrade to neat chitosan hydrogels [84] (Figure 5). The printability of chitosan, including ion complexes (e.g., chitosan–alginate), was well documented, and 3D-printed chitosan scaffolds are attractive scaffold materials for CTE [71,73,91,92,93].

Chitosan–hyaluronic acid complexes were studied and resulted in promising CTE materials showing good mechanical properties and excellent growing ground for bovine articular chondrocyte cells [87]. The addition of hyaluronic acid to ion complexes improves the ability of the complexes to form stable hydrogels. Furthermore, it provides the ion complex with several medical benefits from improving lubrication of articulating surfaces, and thus reducing joint pain, to anti-inflammatory effects and inhibitory effects on prostaglandin synthesis and proteoglycan release and degradation [94].

#### 3.1.4. Hydrogels from Cellulose Derivatives for CTE

Cellulose is the most abundant polysaccharide in nature. It can be extracted from plants, natural fibers, or bacteria. Cellulose hydrogels can be produced from native cellulose solution via hydrogen bonds or water-soluble cellulose derivatives (e.g., methylcellulose (MC), carboxymethyl cellulose (CMC), etc.) via physical or chemical crosslinking. The 3D bioprinting of human nasoseptal chondrocytes with nanocellulose–alginate bioink was investigated for CTE applications [66]. Recently, human-derived induced pluripotent stem cells (iPSCs) were shown to be 3D bioprinted into cartilage using a nanofibrillated cellulose (NFC) composite bioink. Two different bioinks, NFC-alginate and NFC-HA, were found suitable for bioprinting iPSCs to support cartilage [75].

Another biocompatible hydrocarbon polymer commonly used for scaffold fabrication is MC due to its high hydrophilicity and water absorption, which is important for nutrient delivery to cells. Unlike cellulose (and nanocellulose, microfibrillar cellulose), MC is soluble in aqueous media. This results from methoxy groups within MC, disturbing the hydrogen bonds, allowing water molecules to enter the polysaccharide structure and bind to the polar side chains electrostatically [95]. Hence, MC can act as a “support ink” for printing (i.e., sacrificial or fugitive ink). The gelation process of MC is fully reversible, and MC is water-soluble in a non-gelled state. Recent studies showed MC’s great potential as a supportive biomaterial that can be utilized in various ways to enable biofabrication, especially extrusion-based bioprinting of bioinks [96,97]. The printing parameters can be optimized to produce MC-based hydrogels for cell sheet engineering or cell delivery applications with controlled and complex-shaped geometries [98]. In CTE, MC was successfully used as a component of an alginate–MCM bioink for bioprinting of embedded bovine primary chondrocytes (BPCs) [76]. Similarly, 3D bioprinting of an alginate–MC blend hydrogel was shown to improve adhesion between printed layers [99].

**Figure 6:**
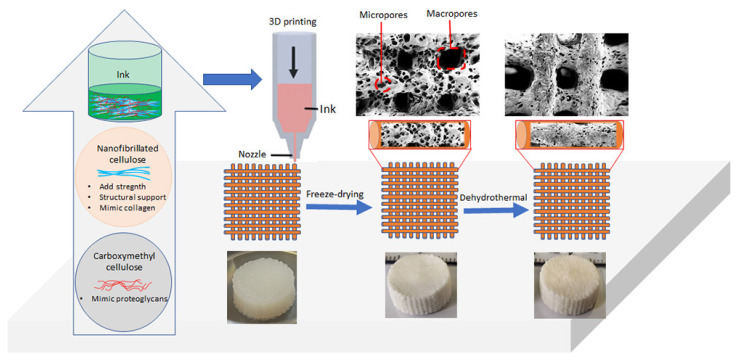
Development of self-standing and lightweight 3D bioscaffolds with microporous and interconnect microporous morphology from bicomponent ink containing NFC and CMC [102], Further permission related to the material excerpted should be directed to the ACS (https://pubs.acs.org/doi/full/10.1021/acsabm.9b01099 (accessed on 8 May 2021)).

Several studies were conducted using CMC to form hydrogels for tissue engineering. For example, a novel hybrid hydrogel was designed using sodium alginate with CMC. This study performed a systematic quantitative characterization and proved its printability, shape fidelity, and cell viability [100]. Another study showed that an amidic derivative of CMC could be considered as a potential compound for cartilage regeneration [101]. Self-standing dual porous 3D bioscaffolds from CMC reinforced with cellulose nanofibrils (CNF) were recently prepared, and its long-term mechanical and dimensional stability in biofluids was shown [102] (Figure 6).

#### 3.1.5. Hyaluronic Acid (HA) Hydrogels for CTE

Hyaluronic acid (HA) is a non-sulfated glycosaminoglycan (GAG) in the ECM of many soft connective tissues, comprised of N-acetylglucosamine and glucuronic acid. Numerous desirable properties of HA, such as high viscoelasticity, biocompatibility, non-immunogenicity, and promotion of cartilage differentiation, make it a suitable carrier material for cell encapsulation and transport of bioactive molecules [103]. HA-based hydrogels have demonstrated an important role in CTE since they induce chondrogenesis by expressing chondrogenic related genes (aggrecan, Sox9, and collagen type 2) and deposition of cartilage-specific ECM components [77]. HA-based hydrogels support chondrogenic differentiation of mesenchymal stem cells (MSCs). Moreover, when compared to PEG hydrogels, HA hydrogels provide a more robust MSCs chondrogenesis and cartilaginous matrix formation both in vitro and in vivo [104]. Several strategies based on the modification of HA have allowed the development of HA formulations suitable to be used as a bioink [78]. The 3D printable HA-based hydrogels showed stable rheology properties and excellent biocompatibility in tissue engineering approaches [48].

**Figure 7:**
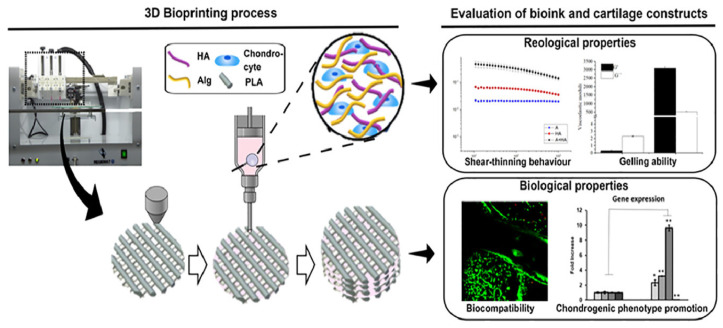
Bio-inspired hydrogel composed of hyaluronic acid and alginate as a potential bioink for 3D bioprinting of articular cartilage engineering constructs [78].

However, HA hydrogels are limited in their applications as cartilage repair scaffolds since they do not provide adequate mechanical properties to remain intact. Therefore, the grafting of synthetic polymers to HA was often performed to provide the necessary stability [48]. Co-printing with natural reinforcing polymers is also performed. An HA–Polylactic acid (PLA) co-printed hydrogel increased the expression of chondrogenic gene markers and specific matrix deposition, thus promoting tissue regeneration [78] (Figure 7). Furthermore, HA is relatively expensive in pure form, which is necessary for medical applications, compared to other PS mentioned above. Therefore, HA was combined with other polysaccharides or proteins to form copolymer hydrogels for CTE. Successfully used composite hydrogels include a chitosan–HA hydrogel [80], a chitosan–HA-based biomimetic matrices [74], HA–agarose Hydrogel [79] and fibrin–HA hydrogel [105]. The addition of hyaluronic acid to polysaccharide-based ion complexes improves the ability of the complexes to form stable hydrogels. Furthermore, it provides the ion complex with several medical benefits from improving lubrication of articulating surfaces and thus reducing joint pain to anti-inflammatory effects, and inhibitory effects on prostaglandin synthesis and proteoglycan release, and degradation [94].

#### 3.1.6. Dextran Hydrogels for CTE

**Figure 8:**
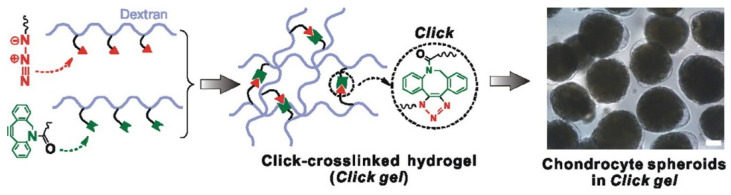
Injectable dextran-based hydrogels crosslinked by metal-free click chemistry [110].

Dextran is a bacterial polysaccharide consisting essentially of α-1,6 linked glucopyranoside residues with a small percentage of α-1,3 linked residues. It is highly water-soluble and very stable under mild acidic and basic conditions. This polysaccharide is used in many biomedical applications due to its biocompatibility, low toxicity, relatively low cost, simple modification, and slow degradation rates by human enzymes as compared to other polysaccharides. In medicine, dextran is used as a plasma volume expander, peripheral flow enhancer, antithrombotic agent, macromolecular carrier for delivery of drugs and proteins, and ingredient in artificial tears [106]. In CTE, dextran hydrogels were shown to be promising for 3D scaffolds applications: e.g., dextran–tyramine conjugates [107], heparin–tyramine conjugates [108], polysaccharide hybrids consisting of HA grafted with a dextran–tyramine conjugate [109], and injectable dextran-based hydrogels [110] (Figure 8).

## 4. Cell Source for Cartilage Tissue Engineering (CTE)

Choosing a suitable cellular source greatly influences the outcome of tissue engineering. CTE is not different, and for its success, it is essential to perform a systematic characterization of the cell source materials, their quality, cellular yield, and chondrocyte proliferation rates. Different cellular sources can be utilized in CTE. Chondrocytes can be derived from animals (mouse, rat, rabbit, bovine) or human cartilage. In both cases, the resulting cells can be planted directly onto scaffolds and are “programmed” to produce cartilage-specific type 2 collagen and related extracellular matrix proteins (e.g., aggrecan) [50,62,64,66,76]. Chondrocytes are typically obtained from the entire thickness of cartilage tissue from the non-load bearing surfaces of articular cartilage and cultured in vitro. On the other hand, human bone marrow-derived mesenchymal stem cells (BMSC) [49], human-derived induced pluripotent stem cells (iPSCs) [75], infrapatellar fat pad adipose stem cells (IPFP-ASCs) [72], adipose-derived stem cells (ADSC) [74], synovial derived stem cells (SDSC) and others [111,112] are also widely used in CTE. Some of these were also already used in combination with polysaccharides-based hydrogels (as seen from Table 1). Although MSCs are generally considered to have a limited potential to undergo chondrogenesis both in vivo and in vitro, they can differentiate into chondrocytes under in vivo conditions, stimulated by the signals arising from the surrounding microenvironment [113].

Microfracture or bone marrow stimulation relies on creating a passageway between the joint space and the underlying bone marrow that allows migration of MSCs into the cartilage defect. Even with direct access to MSCs, this technique typically results in the formation of fibrocartilage, which often temporarily relieves clinical symptoms but does not represent the correct articular cartilage phenotype. In addition, the local microenvironmental conditions at the defect may compromise the natural healing ability of MSCs and their differentiation potential [114]. Similar to these observations, bone marrow-derived MSCs (BM -MSCs) can form a cartilage-like tissue in vitro under the guidance of a specific cocktail of growth factors. In such differentiated cells (and the ECM formed), the collagen content is usually less than 50% of what is found in native healthy adult cartilage, which has a negative impact on tensile strength and load capacity [114]. One of the possible explanations for the impaired outcome of Autologous Chondrocyte Implantation (ACI) is that chondrocytes lose their cartilage-like properties during in vitro expansion and enter a dedifferentiation state [115], leading to the formation of fibrocartilage, with less satisfactory long-term mechanical durability [116].

## 5. Further Consideration—Crucial Testing Methods for an Effective Translation from CTE Research to Clinical Practice

Although a wide variety of scaffolds have been used in CTE in vitro, a clinical translation of these technologies for cartilage repair and regeneration remains a challenge. A limited cell availability, expansion potential, loss of cartilage phenotype, and the absence of the in vivo environmental stimuli are serious drawbacks of CTE research and development in vitro. Therefore, chemical agents (e.g., growth factors) and mechanical stimuli using bioreactors (e.g., dynamic compression, hydrostatic pressure, and tension) have been applied as effective chondro-differentiation agents during the in vitro studies. Thus, tissue maturation can be established, and simulation of the in vivo conditions can be achieved to some extent. Nevertheless, in vitro testing remains an essential step in CTE scaffold development. It is cost-effective compared to the in vivo animal models and allows the characterization of scaffold relevant architectural features, verification of its biocompatibility, cytotoxicity, and ability of phenotype preservation [117,118].

For translation to humans, an in vivo culture in preclinical animal models is mandatory. The latter allows additional assessment of the engineered construct on the biocompatibility, safety, degradation, and tumorigenicity, and focuses on the cellular component and phenotype. Both small and large animal models should be used to test the safety, efficacy, and durability of response in the development of new regenerative strategies in CTE. Small animal models (e.g., rabbit) can provide initial information about safety and efficacy of novel regenerative strategies, whereas a large animal model (e.g., sheep, horse) is necessary to further evaluate efficacy for load-bearing joints such as the knee [119,120,121]. Finally, novel treatment strategies in cartilage regeneration require evaluation via multiple small and large animal models before first human trials. Thus, it must be encouraged to publish the methods and results of any veterinary product transparently to accelerate and ultimately facilitate the establishment of novel CTE strategies for humans.

The scaffold (bio)degradation is another important characteristic in CTE since it affects not only the cartilage tissue formation but also its function. The process of degradation depends on several factors, e.g., type of material and chemical composition (natural, synthetic, or composite scaffolds), surface properties and architectural features (pore size, interconnectivity, porosity), environmental factors (in vivo or in vitro culturing), etc. An ideal hydrogel scaffold should degrade (and/or remodel) at a rate matching tissue regeneration to avoid collapse of the tissue due to premature degradation or cell inflammation and excessive regeneration in the period of delayed degradation [122]. During the initial implantation phase, the scaffold must provide mechanical support for cells to attach and grow. Later, when the scaffold degrades, this function is replaced by cells that migrate into the interior of the scaffold, where they produce ECM and form regenerated tissue [25,123]. Overly rapid degradation may lead to the reduced retention of ECM proteins, whereas slow degradation may hinder cell remodeling and tissue formation. Previous studies have shown that a balanced biodegradation rate promotes neocartilage or bone tissue formation and achieves better mechanical properties after long-term culture [124]. Naturally derived polysaccharides have the advantages of good biocompatibility and biodegradability compared to synthetic hydrogels. Among the main reasons are also the by-products or residual monomers of synthetic scaffolds that might be harmful in the interaction with cells and tissues. In addition, copolymers also have limited biodegradability because of the presence of a non-biodegradable part of the polymer [125,126].

Tracking hydrogel-based biomaterial degradation in vitro and in vivo is mandatory for the rational design of tissue engineering scaffolds in CTE. In vitro, the kinetic equation of hydrogel degradation is obtained by weight measurement and visualization process and can predict in vivo degradation. In general, the degradation rate of scaffolds in CTE is the fastest in the first weeks (weight loss up to 50%) and stabilizes as shown in experiments up to 12 weeks [31,74]. However, in vitro models, which simulate potential hydrolytic, oxidative, and enzymatic degradation, are rarely adequate measures of implant behavior since the degradation in vivo is far more complex. The strategy for monitoring in vivo degradation in real-time in a non-invasive manner could be applied with the help of fluorescence-related visual imaging to track subcutaneous degradation of injectable hydrogels [122,127].

## 6. Conclusion and Future Perspective of 3D Printing of Polysaccharide–Protein-Based Hydrogels

As can be seen from the recent developments in CTE, polysaccharides are among the most commonly applied base material, owing to their excellent intrinsic characteristics. These include their high abundance, tailored physicochemical properties, and optimal biocompatibility with either stem cells that can be transformed into chondrocytes or chondrocytes directly. In this review, we have focused on their use to prepare hydrogels, which can then be used for 3D (bio) printing of either in vitro tissue models or direct products to be used in regenerative medicine. Although a lot of research was already performed in this field, there are still many obstacles to be overcome to allow an effective translation of research into clinical practice. Among these, the most important seems to be tailoring the mechanical properties of these hydrogels with various additives without affecting the biocompatibility of the base materials. Thus, the combination of polysaccharides and proteins (proteo-saccharides) coupled with 3D (bio) printing technologies presents a logical strategy for scaffold development since it resembles ECM structure and guides cell growth and proliferation.

Furthermore, this review shows that most of the available research studies focused only on some aspects of translation into practice. Most do not include all the necessary steps in assessing safety and efficiency in cell or animal models. Keeping this in mind, we believe that systematic studies that include rigorous material development and testing, followed by all levels of safety and efficiency evaluation (i.e., on cell models, in small and big animals), will be necessary to push this field further towards effective and applicable solutions.

## Figures and Tables

**Table 1:** Natural derived polysaccharides hydrogels and their combination with proteins in CTE.

Hydrogels	Cell Type	Scaffold Formation	Main Features
Alginate [32,54]	*various*	Alginate-based bioinks	biocompatibility, degradability, process flexibility and excellent printability
Fibrin [62]	hBM-MSC	Blended hydrogel	promoted cell proliferation
Gelatin and Nhap [31]	Mouse chondrocytes	3D printed hydrogel	high cell viability, supported cellular adhesion and growth
Gelatin and fibrinogen [49]	Human bone MCS	3D bioprinting	chondrogenic differentiation, ECM synthesis, chondrogenic phenotype
Fibrin and HA [50]	Rabbit chondrocytes	3D bioprinting bioinks	proper environment for cartilage formation
Collagen [64]	Rats’ chondrocytes	3D bioprinting bioinks	cell adhesion, proliferation and expression of cartilage specific genes
Nanocellulose [65]	Articular cartilage (calves)	Printable bioink	promoted cell spreading, proliferation, and collagen II synthesis by the encapsulated cells
Agarose [28,69]	*various*	Agarose-based hydrogels	biocompatibility, water solubility, adaptable mechanical properties, printability
Silk Fibroin [70]	Cartilaginous tissue	Blended hydrogels	immunocompatibility, deposition of glycosaminoglycans (GAG) and collagen, upregulation of cartilage genes
Fibrin [51]	HECDC	Nanostructured hydrogels	biodegradable and biologically active constructs
Chitosan [71,72],	Chondrocyte, IFP-ASCs	3D-printed hydrogels	biocompatibility, cellular morphology, mechanical properties, chondrogenesis
CM Chitosan [73]	Rabbit chondrocytes	3D bioprinting bioinks	cell attachment, favorable mechanical property, chondrogenic gene expression
Chitosan-HA [74]	ADSC	Biomimetic Matrices	supports stem cell differentiation towards cartilage matrix producing chondrocytes
Cellulose			
NFC-Alginate [66]	human chondrocytes	3D bioprinting	potential use of nanocellulose for 3D bioprinting of living tissues and organs
NFC-Alginate and HA [75]	iPSCs	3D bioprinting	NFC/A bioink is suitable for bioprinting iPSCs to support cartilage production
Methylcellulose (MC)			
Alginate-MC [76]	bovine chondrocytes	Bioink for bioprinting	3D-printing-based fabrication, bioengineered tissue for cartilage regeneration
Hyaluronic acid [77,78],	*various*	Hydrogels	stimulates the chondrogenic differentiation, produce essential cartilage ECM
Alginate-HA [78]	hAC	HA-based bioink (hydrogel)	cell functionality, expression of chondrogenic gene markers, specific matrix deposition
Agarose-HA [79]	rabbit chondrocytes	Hydrogels	improved viability, proliferation, morphology and adhesion of the chondrocytes
Chitosan-HA [80]	rabbit chondrocytes	Hydrogels	in vivo study (rabbits); implant had a mixture of hyaline and fibro cartilage
Chitosan-HA [74]	ADSC	Biomimetic Matrices	supports stem cell differentiation towards cartilage matrix producing chondrocytes
Gelatin-HA [52]	hBMSCs	Hybrid hydrogel	in vivo study (rabbit femoral condyle) promising scaffold for repair and resurfacing
Collagen-HA [53]	rabbit	Hybrid scaffolds	in vivo study (cartilage defects of rabbit ear)

hBM-MCS/hBMSCs—human bone marrow stem cells, NFC—nanofibrillated cellulose, HECDC—encapsulating human elastic cartilage-derived chondrocytes, ADSC—adipose-derived stem cells, CM—carboxymethyl, IPFP-ASCs—infrapatellar fat pad adipose stem cells, iPSCs—human-derived induced pluripotent stem cells, hAC—human articular cartilage.

## Data Availability

No new data were created or analyzed in this study. Data sharing is not applicable to this article.
